# HSD17β11 regulates PLIN5-ATGL mediated lipolysis, but not hepatic lipid metabolism in mice

**DOI:** 10.1016/j.jlr.2025.100943

**Published:** 2025-11-12

**Authors:** Stacey N. Keenan, Natasha D. Suriani, Gio Fidelito, Jackie Bayliss, Jieqiong Lou, Ashleigh N. Solano, Joanna Sacharz, David A. Stroud, Ayenachew Bezawork-Geleta, Geraldine Ooi, Paul R. Burton, Elizabeth Hinde, Matthew J. Watt

**Affiliations:** 1 Department of Anatomy and Physiology, School of Biomedical Sciences, Faculty of Medicine, Dentistry and Health Sciences, The University of Melbourne, Melbourne, Victoria, Australia; 2 School of Physics, University of Melbourne, Melbourne, Victoria, Australia; 3 Department of Biochemistry and Pharmacology, School of Biomedical Sciences, Faculty of Medicine, Dentistry and Health Sciences, Melbourne, Victoria, Australia; 4 Murdoch Children’s Research Institute, Melbourne, Victoria, Australia; 5 Victorian Clinical Genetics Services, Murdoch Children’s Research Institute, Melbourne, Victoria, Australia; 6 Department of Surgery, School of Translational Medicine, Monash University, Bariatric Unit, Department of General Surgery, The Alfred Hospital, Melbourne, Victoria, Australia

**Keywords:** lipid metabolism, lipolysis, PKA, phosphorylation, lipid droplet, metabolic dysfunction-associated steatotic liver disease

## Abstract

Hydroxysteroid 17β dehydrogenase 11 (HSD17β11) is a member of the 17β-HSD family with canonical roles in steroid metabolism. Given its predominant localization on lipid droplets, we investigated HSD17β11's role in lipid metabolism. In patients with metabolic dysfunction-associated fatty liver disease (MASLD), liver HSD17β11 levels are reduced, correlating with liver steatosis severity. HSD17β11 deletion in human cell lines increased lipid droplet size and number. This is associated with triglyceride accumulation due to impaired lipolysis and increased fatty acid uptake. Mechanistically, HSD17β11 facilitates the interaction between PLIN5 and ATGL, enabling efficient protein kinase A (PKA)-stimulated lipolysis. Surprisingly, *Hsd17β11* deletion did not affect liver lipid metabolism or MASLD development in lean or obese mice. These findings demonstrate that while HSD17β11 is crucial for efficient PKA-mediated lipolysis in human cells, its deficiency appears redundant for lipid metabolism in mice.

## Graphical abstract

**Figure:**
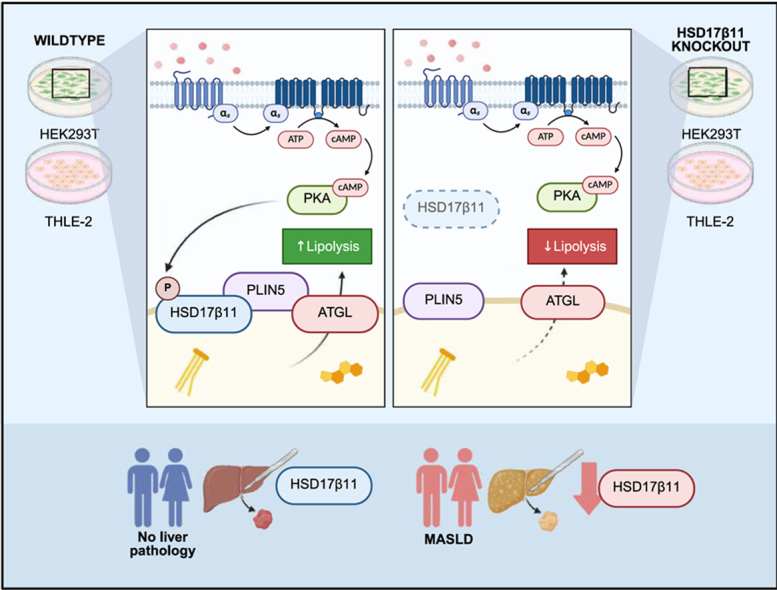

Lipid droplets (LDs) are highly dynamic organelles that engage in extensive interactions with other cellular compartments to regulate lipid homeostasis. Under conditions of nutrient excess, LDs sequester surplus fatty acids into neutral lipids, serving as a protective buffer against lipotoxicity and oxidative stress. Conversely, during nutrient deprivation, fatty acids are released to support mitochondrial ATP production (1). Proteins of diverse functions localize to the surface of LDs, and these can be stably associated with LD membranes, most commonly via the insertion of a hydrophobic hairpin, or recruited directly from other cellular locations to the LD surface (2). Advancements in organelle-specific isolation techniques and proximity-based labelling approaches, coupled with mass spectrometry-based proteomics, have revealed a remarkably complex LD-associated proteome comprising thousands of proteins across diverse cell types (3, 4, 5). Notably, enzymes involved in lipid metabolism constitute the core of this proteome, underscoring the LD’s central role in cellular lipid regulation (6). Elucidating the composition and dynamic interactions of LD-associated proteins has been instrumental in advancing our understanding of the spatial and temporal control of triglyceride storage and lipolysis in cells.

HSD17β11 was initially identified as a bona fide LD-associated protein in immortalized human hepatocytes (Huh7 cells) (7), and subsequent studies have confirmed its enrichment on LDs in many cell types (3, 8, 9). As a member of the 17β-hydroxysteroid dehydrogenase (17β-HSD) enzyme family, HSD17β11 belongs to a group of oxidoreductases primarily recognized for their roles in steroidogenesis and steroid metabolism. However, emerging evidence indicates that HSD17β11 also participates in broader aspects of lipid metabolism (10). Functional studies in *Caenorhabditis elegans* have shown that loss-of-function mutation of the HSD17β11 ortholog leads to reduced LD aggregation and lower intracellular triglyceride levels (11), suggesting a conserved role in LD biogenesis and/or triglyceride turnover. Consistent with this, overexpression of HSD17β11 in HeLa cells promotes intracellular triglyceride accumulation (11), further implicating this protein in lipid storage regulation.

Mechanistic insights into HSD17β11 localization have revealed that its N-terminal region is critical for targeting to LDs. Specifically, amino acids 1–28 are necessary for LD association, and deletions within this region, particularly residues 4–16 or the patatin-like domain spanning residues 22–28, result in mislocalization, indicating that multiple N-terminal motifs contribute to correct subcellular targeting (12). More recently, HSD17β11 was shown to undergo ubiquitination in response to ethanol exposure, with subsequent proteasomal degradation implicated in the regulation of ethanol-induced hepatic steatosis (13). Collectively, these findings highlight HSD17β11 as a multifaceted regulator of LD metabolism. Nonetheless, the precise molecular mechanisms by which HSD17β11 influences LD dynamics and triglyceride flux remain incompletely defined, warranting further investigation.

Among the 17β-HSD enzyme family, HSD17β11 and HSD17β13 are uniquely localized to LDs in mammalian cells. HSD17β13 exhibits highly liver-specific expression and has garnered significant attention due to its association with a protective loss-of-function splice variant (rs72613567:TA) linked to reduced risk of metabolic dysfunction-associated steatohepatitis and progression to cirrhosis (14, 15). Notably, HSD17β11 shares 78% sequence homology with HSD17β13, and both genes are colocalized within a conserved chromosomal locus in human, mouse, and rat genomes (16). Their high degree of structural similarity (17) suggests an evolutionary relationship and potentially overlapping functions.

Here, we demonstrate that HSD17β11 acts as a mediator of PKA-stimulated lipolysis by orchestrating protein-protein interactions with ATGL and perilipin 5 (PLIN5) at the LD surface. These findings position HSD17β11 as an important effector in the regulation of LD-associated lipid mobilization in vitro. Further studies in whole-body knockout mice reveal no notable changes in liver or whole-body lipid metabolism. This unexpected result suggests that HSD17β11 may be redundant in vivo, possibly due to compensatory upregulation of HSD17β13.

## Materials and methods

### Human studies

Participants provided written and verbal informed consent. The study protocol conforms to the ethical guidelines of the 1975 Declaration of Helsinki and was approved by the Human Research Ethics Committee of participating sites (Alfred (195/15), Avenue (190) and Cabrini (09-31-08-15) Hospitals, Melbourne, Australia) and registered with the Australian Clinical Trials Register (ACTRN12615000875505). We prospectively enrolled consecutive eligible patients with severe or morbid obesity undergoing bariatric surgery. Inclusion criteria included: age > 18 years, BMI > 35 kg/m^2^, alanine aminotransferase (ALT) or aspartate aminotransferase (AST) > 0.5 times the upper limit normal (ULN), or gamma-glutamyl transferase (GGT) > ULN. Exclusion criteria included: evidence of other liver disease, including viral hepatitis, medication related, autoimmune, familial/genetic causes or a history of excessive alcohol use. Intraoperative wedge liver biopsies, 1 cm in depth, were taken. Steatosis severity by image analysis: Steatosis was measured as the percentage area occupied by LD vacuolization on H&E-stained liver sections, measured using Fiji ImageJ image analysis. These data were used to report percentage changes in lipid species with every 1% increase in area of histological steatosis. Pathologist-defined MASLD: A single pathologist graded the biopsies in a blinded manner. Patients were stratified into groups based on pathology’s assessment: “No-MAFL” - no significant steatosis; non-MASH MAFL (“MAFL”) - any degree of steatosis, without significant inflammation; non-alcoholic steatohepatitis (“MASH”) - joint presence of steatosis with significant inflammation (at least 1 point inflammation and 2 points ballooning, or vice versa) as previously defined (18, 19). Liver fibrosis was graded according to the Kleiner classification. Participants who did not fall into these categories were excluded from this categorical analysis.

### Generation of cell lines

HEK293T HSD17β11 knockout cells were generated by CRISPR-Cas9 gene editing using oligonucleotides encoding gRNA sequence (forward primer: 5′-CACCGGGCGAAATCGTGCTGATTAC, reverse primer: 3′-AAACGTAATCAGCACGATTTCGCCC) were cloned into pSpCas9(BB)-2A-GFP (PX458) plasmid (gifted from F. Zhang; Addgene, #48138). Constructs were transfected with Lipofectamine LTX (ThermoFisher Scientific) according to manufacturer’s instructions. Single GFP+ cells were sorted and clonal populations were expanded and screened for HSD17β11 knockout by SDS-PAGE and immunoblotting. Candidate HSD^KO^ clones then had genomic DNA isolated using Quick-DNA kit (Zymo Research, #D3024) according to manufacturer’s instructions to validate CRISPR-Cas9-induced insertions and deletions.

THLE-2 HSD17β11 knockout cells were generated by transducing Lenti-guide-puro (Addgene, #52963) to Cas9 expressing THLE-2 cells (THLE-2 Cas9), previously generated with FUCas9Cherry vector (Addgene, #70182). The sgRNA sequences were NTG (GTATTACTGATATTGGTGGG), HSD17β11 knockout-1 (GGCGAAATCGTGCTGATTAC), HSD17β11 knockout-2 (CGATCAGTAACGGGAGAAGC). Lentiviral particles were generated using the third-generation packaging system by transiently transfecting pMDLg/pRRE (Addgene, #12251), pRSV-REV (Addgene, #12253), pVSVG (Addgene, #14888), and transfer plasmid into HEK-293T cells using the polyethylenimine (PEI) reagent, as previously described (20). THLE-2 Cas9 cells were then incubated with lentivirus supplemented with polybrene (8 μg/ml) overnight before media was replenished with fresh media. Transduced cells were selected with puromycin (3 μg/ml).

HSD17β11-FLAG cells were generated by stable re-expression of FLAG-tagged WT HSD17β11 (herein denoted HSD^FLAG^) in HEK293T HSD^KO^ cells. HSD^FLAG^ oligonucleotides were purchased from Sigma-Aldrich and used to amplify HSD17β11 cDNA with the resulting product subcloned into pBABE-puro (Addgene, #1764). Plasmids were then purified and were validated by Sanger sequencing (AGRF). Retroviral particles were generated using a second-generation packaging system by transiently transfecting pUMVC (Addgene, #8449), pCMV-VSV-G (Addgene, #8454) and the transfer plasmid pBABE-puro encoding HSD^FLAG^ into HEK293T cells. Retroviral particles were then used to transduce HSD^KO^ HEK293T cells followed by selection with puromycin.

### Lipid droplet staining and quantification

As previously described (21), HEK293T were plated and cultured in chambered coverslips (Sarstedt, Germany) and treated with or without 20 μM forskolin for 2 h. Culture medium was then incubated with 2 μmoL/l BODIPY™ FL C16 (ThermoFisher Scientific) for 15 min. Cells were fixed in 4% paraformaldehyde for 15 min at room temperature, washed with DPBS and stained with 4′,6-diamidino-2-phenylindole (DAPI) (ThermoFisher Scientific) (1 μg/ml) to visualize nuclei. Cells expressing FLAG-tagged proteins were probed with anti-FLAG antibody (Sigma, #F9291) followed by anti-mouse Alexa Fluor™ 647 (ThermoFisher Scientific). Following secondary incubation, cells were washed and mounted on glass slides as described previously. Cells were viewed using confocal microscopy (ZEISS LSM 900 with Airyscan 2, Germany) at 63X objective (oil-immersion). LD number and area was quantified using Fiji (ImageJ) software version 1.8.0_112 (National Institutes of Health).

### Assessment of fatty acid metabolism

Cells were lipid-loaded for 12 h in low-glucose DMEM GlutaMAX containing 500 μmol/L palmitate:oleate (2:1 M ratio) conjugated to 2% fatty-acid-free BSA before commencement of radiometric analysis. Cells were incubated for 2 h in low-glucose DMEM containing 500 mM oleic acid and 1 mCi/ml 1-^14^C-oleic acid (GE Healthcare). Medium was acidified in 1M perchloric acid, CO_2_ captured in 1 M NaOH, and then radioactivity was counted using a liquid scintillation counter (Tri-Carb 4910 TR 110 V Liquid Scintillation Counter; PerkinElmer). Lipids were extracted in 2:1 (v/v) chloroform: methanol, phase separation initiated by the addition of 0.9% NaCl, the aqueous layer was used for assessment of acid-soluble metabolites, and the lower organic phase was transferred to a fresh tube, dried under N_2_ at 40°C, then reconstituted in 2:1 chloroform: methanol containing lipid standards for cholesterol ester, triglyceride, diglyceride, ceramide, and phospholipid (Sigma). The lipid mixture was spotted onto a glass-backed Silica Gel 60 plate, and the lipids were resolved. The plates were air-dried, sprayed with dichlorofluorescein (0.02% w/v in ethanol) dye, and the lipid bands were visualized under UV light. The lipid bands were scraped, and radioactivity incorporation was assessed by lipid scintillation counting. Fatty acid uptake was calculated as the sum of fatty acid oxidation and fatty acids stored in complex lipids (i.e., ^14^C in the organic fraction of the lysed cells). All values were normalized to total cellular protein (BCA method, ThermoFisher Scientific).

To assess lipolysis, “pulse-chase” experiments were conducted. Cells were “pulsed” in low-glucose DMEM containing 500 μmol/L palmitate and 1.5 μCi/ml [1–^14^C] palmitate conjugated to 2% BSA for 16 h. This leads to ∼85% of all ^14^C-palmitate contained in cellular lipids being incorporated into triglycerides (22). At the end of the “pulse” period, half of the cells were washed 3 times with ice-cold PBS and lysed as described previously. A portion of these cells was subjected to biochemical determination of triglyceride as described below. Adjacent cells were washed 3 times with warm PBS to remove extracellular ^14^C palmitate and incubated in “chase” media containing 20 μmol/L forskolin, 6 μmol/L Triacsin C (Enzo Life Sciences, #BML-EI218), 1 mmol/L L-carnitine in low-glucose DMEM for 6 h. Cells were washed in PBS and lysed as described above. ^14^C incorporation in triglyceride was determined by resolving the spotted lipid mixture onto a glass-backed Silica Gel 60 plate, and the triglycreride band was scraped for scintillation counting of both the ‘pulsed’ and ‘chased’ cells. Lipolysis was calculated as the difference in ^14^C incorporation into triglycerides between ‘pulse’ and ‘chase’ normalized to unlabeled triglyceride content.

### RNA isolation and quantitative polymerase chain reaction

RNA was isolated from cells using TRI-Reagent (Sigma–Aldrich), treated with DNAse (Ambion DNA free kit, Thermo Fisher) and reverse transcribed into cDNA with iSCRIPT Reverse Transcriptase (Invitrogen) as per the manufacturer's instructions. Real-time PCR was performed using the SYBR Green PCR master mix (Quantinova® SYBR Green PCR kit, QIAGEN) and expression was determined using a CFX Connect™ Real-Time PCR Detection System (Biorad). All samples were normalized using the housekeeping gene HPRT, and primer sequences are provided below. The mRNA levels were analyzed by the 2^−ΔΔCT^ method. Primer sequences are listed in Supplemental Table S2.

### Immunoblotting

Cell lysates were prepared in RIPA buffer, proteins were resolved by SDS-PAGE electrophoresis, and immunoblot analysis was conducted. Stain-free images were collected after transfer to correct for loading differences across samples (ChemiDoc MP and ImageLab software Version 4.1, Bio-Rad Laboratories). The membranes were probed with antibodies raised against HSD17β11 (17301-1-AP, Protein Tech, 1:1000 dilution in TBST containing 2.5% skim milk and 2.5% BSA), HSD17β13 (OAAN01691, Aviva System Biology, 1:1000 dilution in TBST containing 5% skim milk) or PLIN5 (GP31, PROGEN, 1:1000 dilution in PBS containing 2.5% skim milk and 2.5% BSA). Data are presented as the density of the immunoreactive band relative to total protein loading for that specific lane.

### FLIM-FRET analysis of protein-protein interactions

For FLIM-FRET experiments, cells were plated onto 35 mm glass-bottom dishes and transiently transfected with Lipofectamine 3000 (Invitrogen™, #L3000001) using the following plasmids: ATGL-CFP, PLIN5-CFP, PLIN5-YFP or HSD17β11-YFP. All FLIM-FRET data were acquired with an Olympus FV3000 laser scanning microscope coupled to a 440 nm pulsed laser operated at 80 MHz and an ISS A320 FastFLIM box for time-resolved detection. All cells were imaged at 37°C in 5% CO2. A 60x water immersion objective (1.2 NA) was used for all experiments. The fluorescence signal was first separated from laser light with a 440 nm dichroic mirror, then directed through a 518 nm long pass filter that split the donor and acceptor signal between two photomultiplier detectors (H7422P-40 of Hamamatsu) of the following bandwidth filters: CFP 488/50 and YFP 520/25. The pixel frame size was set to 256 x 256, which gave a pixel size of 104 μm. The pixel dwell time was set at 20 μs/pixel for a 1.61 s frame time. 20 frames were integrated per FLIM experiment. These conditions resulted in an acquisition time of ∼0.5 min. Calibration of the system and phasor plot space was performed by measuring fluorescein (pH 9.0), which has a known single exponential lifetime of 4.04 ns. The FLIM data were acquired by ISS Vista Vision and processed by the SimFCS software developed at the Laboratory for Fluorescence Dynamics (LFD, www.lfd.uci.edu).

FLIM-FRET data were quantified by the phasor approach to fluorescence lifetime analysis as previously published (22, 23, 24). Briefly, the fluorescence decay recorded in each pixel of a FLIM-FRET image is described by a g- and s-coordinate (phasor) in the phasor plot that in reciprocal mode enables each point of the phasor plot to be mapped to each pixel of the FLIM image. In the case of a FRET experiment where the lifetime of the donor molecule is changed upon interaction with an acceptor molecule, the realisation of all possible phasors quenched with different efficiencies describes a curved trajectory in the phasor plot. The FRET trajectory follows the classical definition of FRET efficiency. The contribution of background (i.e. cellular autofluorescence) versus the contribution of the unquenched donor are evaluated using the rule of the linear combination. Acquisition of a FLIM image in the donor channel records the fluorescence lifetime in each pixel, producing a readout of FRET and protein-protein interaction, i.e. pixels with a quenched lifetime undergo FRET. The fraction of pixels that exhibit this quenched lifetime are then quantified as a percentage, as well as the FRET efficiency of this interaction, which is related to how quenched the fluorescence lifetime is, providing a readout of the strength of the protein-protein interaction. The FRET efficiency of each donor-acceptor interaction and phasor location was used to spatially map PLIN5 interaction with HSD17β11 as well as quantify the frequency (i.e., fraction of pixels) of this interaction across multiple cells. All FLIM-FRET quantitation was performed using SimFCS software developed at the LFD.

### Proteomic analysis of interacting proteins

HEK293T cells stably expressing empty vector (3HA-eGFP, control), or 3HA-PLIN5 were grown on 10 cm dishes. Cells were lysed in 0.75 ml lysis buffer (25 mmol/L HEPES pH 7.4, 150 mmol/L NaCl, 1 mmol/L EDTA, 10% glycerol, 1% wt/vol n-dodecyl-β-D-maltoside) on ice for 30 min. The lysates were cleared by centrifuging at 18,000 × g for 15 min at 4°C. An equal volume of cell lysates (1∼2 mg of protein) was mixed with 25 μl EZview™ Red Anti-HA Agarose beads (#E6679; Sigma-Aldrich). The protein bead mixture was gently rotated at 4°C overnight, followed by washing three times with PBS and centrifuging at 16,000 × g for 3 min at 4°C. For proteomic analysis of the immunoprecipitated proteins, the cysteine bonds were reduced on beads with 5 mmol/L Tris(2-carboxyethyl)phosphine (TCEP) for 30 min at 37°C followed by alkylation with 10 mmol/L iodoacetamide. Beads were then resuspended in digestion buffer containing sequencing-grade modified trypsin (Pierce) at 37°C overnight. After quenching with 10% TFA, the samples were desalted by C18 reversed-phase spin columns according to the manufacturer’s instructions (Pierce). The eluted peptide sample was dried in a vacuum centrifuge and reconstituted to a final volume of 30 μl in 0.1% TFA and 1% CH_3_CN. Analysis was performed on a Q-Exactive mass spectrometer (Thermo Fisher Scientific) coupled to liquid chromatography system. The raw files were first searched by Maxquant and enriched proteins were analyzed by Perseus platform using the same search parameter as indicated above.

### Animal experiments

All experimental procedures were approved by the University of Melbourne Animal Ethics Committee (#20824). *Hsd17β11* knockout mice were generated by the Melbourne Advanced Gene Editing Centre laboratory (Walter and Eliza Hall Institute) on a C57BL/6J background. To generate mice, 20 ng/μl of Cas9 mRNA, 10 ng/μl of sgRNAs (ACTGTTCGATCTCGGACCCT and CTGAATAGAGAGTTAATCGT), respectively, were injected into the cytoplasm of fertilized one-cell stage embryos generated from wild-type C57BL/6J breeders. Twenty-four hours later, two-cell stage embryos were transferred into the uteri of pseudo-pregnant female mice. Viable offspring were genotyped by next-generation sequencing. Targeted animals were backcrossed twice to wild-type C57BL/6J to eliminate off-target mutations. Mice were maintained at 22°C on a 12:12 h light-dark cycle and fed ad libitum with access to either chow (5% of energy from fat, Specialty Feeds) or high-fat diet (HFD, 43% energy from fat) (High Fat Rodent Diet SF04-001; Speciality Feeds) and water starting at 10 weeks of age for a total of 12 weeks. Mice were fasted from 0700 to 1100 h before all experiments unless stated otherwise.

### Metabolic cage studies

Mice were individually housed in a Promethion Metabolic Cage System (16 chambers; Sable System International) for 48 h to assess oxygen uptake, carbon dioxide production, daily physical activity and food intake (Melbourne Mouse Metabolic Phenotyping Platform, Department of Anatomy and Physiology, The University of Melbourne). Respiratory exchange ratio was calculated as the ratio of CO_2_ production over O_2_ consumption and energy expenditure was calculated using the Weir equation (energy expenditure (kcal h^−1^) = 60 × (0.003941 × VO_2_ + 0.001106 × VCO_2_). Carbohydrate and fatty acid and oxidation were calculated using the following equations: carbohydrate oxidation (g/min) = (4.55 x VCO_2_) – (3.21 x VO_2_); fat oxidation (g/min) = (1.67 x VO_2_) - (1.67 x VCO_2_). Gases were assessed at 30 min intervals for 48 h following a 12-h acclimatisation period.

### Plasma metabolites

Plasma was isolated by centrifugation of whole blood at 3,000 g at 4°C for 10 min. Plasma cholesterol was assessed by colorimetric assay as per manufacturer's instructions (Wako Diagnostics). Plasma triglyceride levels were evaluated by colorimetric assay as per the manufacturer's instructions (ThermoFisher Scientific, #TR22421). Plasma alanine aminotransferase (ALT) and aspartate aminotransferase (AST) were assessed by enzymatic assay as previously described (25). For each assay, absorbance was measured using a spectrophotometer (SPECTROstar Nano, BMG LABTECH).

### Liver histology

Liver from the same lobe of fasted mice was obtained and fixed in 10% neutral buffered formalin for 24 h before transfer to 95% ethanol solution. Livers were paraffin embedded, cut into 10 μm liver sections, and stained with hematoxylin and eosin (University of Melbourne Histology Platform). Sections were imaged on a BXM53 M light microscope (Olympus) at 10x magnification. LDs were identified using ilastik™ machine learning image analysis (26) and converted into an 8 bit image for quantitation of LD number by ImageJ2 (version 2.9.0). LDs less than 300 μm^2^ in area were considered “true” LDs, based on previous studies (22, 25) and considering liver mass and duration of high-fat feeding. Steatosis score was calculated as the total LD area as a percentage of total liver area.

### Liver triglyceride

Lipids were extracted from 20-30 mg of liver in 1.8 ml 2:1 chloroform:methanol (v:v) and triglyceride content was assessed with Infinity™ Triglycerides Reagent (ThermoFisher Scientific™, #TR22421) assay relative to a glycerol standard (Sigma, #G7793), per manufacturer’s instructions.

### Liver lipid metabolism

Precision-cut liver slices were generated as previously described (22). Following at least 1 h of settling in oxygenated M199 media, the liver slices were washed in PBS and transferred to glass vials containing low-glucose (5 mmol/L) DMEM, 0.5 mmol/L palmitic acid and 1 μCi/ml [1–^14^C] palmitic acid (NEC075H250UC; PerkinElmer) conjugated to 2% BSA for 2 h. Fatty acid oxidation was calculated as the sum of ^14^CO_2_ production and ^14^C-palmitate conversion to acid-soluble metabolites. Lipids were extracted, separated, and incorporation of ^14^C-palmitate into individual lipid species was determined by thin layer chromatography as previously described (22). Fatty acid uptake was calculated by adding fatty acid oxidation to total lipid storage.

### Statistics

All data are presented as mean ± SEM. In comparisons made between two groups, an unpaired two-tailed Student's *t* test was performed. In comparisons with more than two groups, one-way or two-way analysis of variance (ANOVA) was performed with Dunnett, Bonferroni or Sidak post hoc analysis where appropriate. The analysis used for specific experiments is outlined in the figure legends. Statistical significance was established a priori at *P* < 0.05.

## Results

### HSD17β11 expression is decreased in human MASLD and type 2 diabetes

**Fig. 1:**
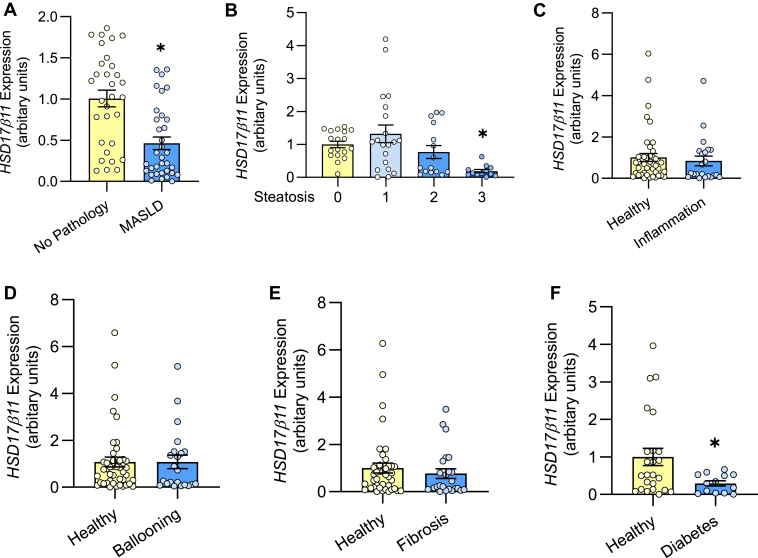
*HSD17β11* mRNA is decreased in human MASLD (A) *HSD17β11* mRNA expression in the livers of patients grouped by no pathology (i.e., no-MASLD; N = 31) and MASLD (N = 34). Liver *HSD17β11* expression according to histological variables including (B) steatosis grade (grade 0: N = 18, grade 1: N = 20, grade 2: N = 15, grade 3: N = 12)), (C) inflammation (N = 22), (D) hepatocyte ballooning (N = 22), and (E) fibrosis (N = 25). F: Liver *HSD17β11* expression in patients with (N = 13) or without type 2 diabetes (N = 24). For all panels, data is presented as mean ± SEM. ∗*P* < 0.05 as assessed by one-way ANOVA and Bonferroni post hoc analysis. MASLD, Metabolic dysfunction-associated steatotic liver disease.

Given its likely role in the regulation of lipolysis and cellular triglyceride levels (11), we first sought to ascertain *HSD17β11* expression in states of excessive lipid accumulation. HSD17β11 is most highly enriched in the liver (27) and dysregulated triglyceride metabolism is a hallmark of hepatic steatosis in metabolic dysfunction-associated steatotic liver disease (MASLD). Accordingly, we assessed HSD17β11 expression in the liver of individuals with normal liver (no pathology) and MASLD. Patient characteristics are described in Supplemental Table S1. Briefly, the male to female ratio was 23:77, with the following characteristics (±SD): age, 45 ± 12.8 years; BMI, 43 ± 7 kg/m^2^; fasting plasma ALT, 35.4 ± 18.6 IU/L; plasma triglyceride, 1.5 ± 0.7 mmol/L; plasma cholesterol, 4.2 ± 1.0 mmol/L; blood glucose, 6.0 ± 1.8 mmol/L; and plasma insulin, 13.2 ± 26.9 mU/L. Patients with MASLD had higher plasma triglycerides (No pathology: 1.1 ± 0.4, MASLD: 1.7 ± 0.8) and ALT (No pathology: 25.5 ± 11.0, MASLD: 41.4 ± 19.8). A single pathologist graded the biopsies in a blinded manner and the patient groups were stratified into “Healthy”, which refers to no significant steatosis, and “MASLD”, which is classified as any degree of steatosis with varying degrees of inflammation, hepatocellular ballooning and fibrosis. Hepatic *HSD17β11* expression was decreased by 52% in individuals with MASLD compared to individuals with no pathology (Fig. 1A). *HSD17β11* expression was decreased by 81% in patients with severe grade 3 steatosis compared with grade 0 steatosis, while there were no differences in expression between grade 0, 1 or 2 steatosis (Fig. 1B). *HSD17β11* expression was not associated with other components of MASLD histopathology including hepatocyte inflammation, ballooning, and fibrosis (Fig. 1C–E). Additionally, *HSD17β11* expression was significantly decreased in individuals with type 2 diabetes (Fig. 1F).

### HSD17β11 regulates lipid droplet size and number and lipolysis

**Fig. 2:**
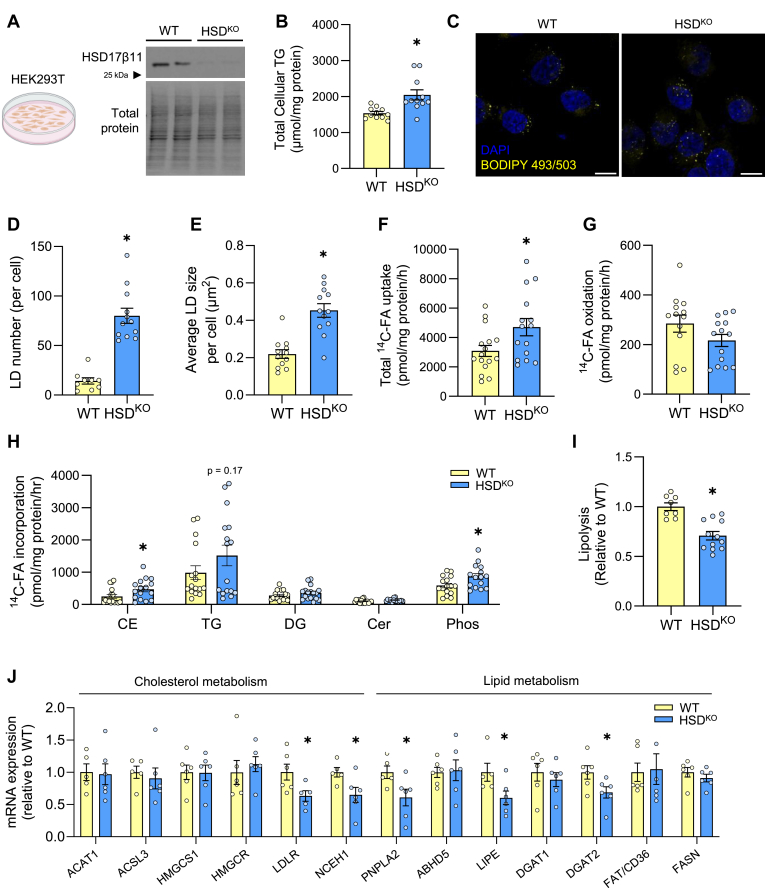
HSD17β11 deletion remodels LD metabolism in HEK293T cells. A: Immunoblot of wild-type (WT) and HSD17β11 knockout (HSD^KO^) cells. B: Cellular triglyceride content in overnight lipid loaded cells (N = 3–4 per group from three independent experiments). C: Representative confocal images of WT and HSD^KO^ cells stained for LDs (BODIPY 493/503) and nuclei (DAPI). Images were quantified to determine (D) LD number (count, per cell) and (E) average LD area (μm^2^). Scale bars represent 40 μm (N = 4–6 per group from two independent experiments). Cells were pulsed with ^14^C-FA to determine (F) uptake, (G) oxidation rate, and (H) incorporation into different lipid species in a 2 h pulse experiment (N = 3–4 replicates from four independent experiments). Cells were pulsed with ^14^C-FA for 16 h followed by a 6 h chase to determine (I) rate of lipolysis (N = 2–4 replicates from three independent experiments). J: mRNA expression of cholesterol and lipid metabolism genes (N = 5–6 replicates from one experiment). Data is presented as mean ± SEM. ∗*P* < 0.05 relative to WT by unpaired *t* test. CE, cholesterol ester; Cer, ceramide; DG, diglyceride; FA, fatty acid, KO, knockout; LD, lipid droplet; Phos, phospholipid; TG, triglyceride; WT, wild-type.

To characterise the role of HSD17β11 in cell metabolism, we generated a HSD17β11 knockout cell line (HSD^KO^) using CRISPR-Cas9 with gRNA targeting the first exon of HSD17β11 in HEK293T cells. Immunoblot analysis revealed an 88% reduction in HSD17β11 protein content in HSD^KO^ compared with wild-type (WT) cells (Fig. 2A). Ablation of HSD17β11 resulted in a 33% increase in cellular triglyceride content compared to WT cells (Fig. 2B). Consistent with this finding, there was a significant increase in both LD number and size in HSD^KO^ compared with WT cells (69% and 92% respectively, Fig. 2C–E), suggesting that HSD17β11 deletion leads to accumulation of triglyceride in LDs.

We next performed fatty acid tracing experiments to investigate the effect of HSD17β11 deletion on cellular lipid metabolism. There was a 49% increase in fatty acid uptake (Fig. 2F) in HSD^KO^ cells and no difference in fatty acid oxidation between HSD^KO^ and WT cells (Fig. 2G). Fatty acid incorporation into cholesterol esters and phospholipids was increased in HSD^KO^ compared with WT cells (92% and 48% respectively, Fig. 2H) while HSD17β11 deletion did not impact fatty acid incorporation into triglycerides, diglycerides or ceramides (Fig. 2H). These data demonstrate that HSD17β11 deletion increases the net flux of fatty acids into neutral lipids and phospholipids in HEK293T cells, without affecting fatty acid oxidation.

Based on its localization to the LD and the lipolytic actions of HSD17β13 (28), we postulated that HSD17β11 plays a role in lipolysis. Results from “pulse-chase” experiments showed that HSD17β11 deletion reduced forskolin-stimulated lipolysis by 29% compared with WT cells (Fig. 2I), highlighting a role of HSD17β11 in lipolysis. This was accompanied by significant reductions in the expression of genes encoding regulatory proteins of lipolysis such as *PNPLA2* (ATGL) and *LIPE* (hormone sensitive lipase, HSL), esterification of fatty acids (*DGAT2*), hydrolysis of cholesterol esters (*NCEH1*, Neutral Cholesterol Ester Hydrolase 1), and lipoprotein metabolism (*LDLR*, LDL-receptor) (Fig. 2J). Collectively, these data show that HSD17β11 deletion alters fatty acid uptake and storage, lipolysis, and the expression of a subset of genes involved in LD synthesis and breakdown.

### HSD17β11 is constitutively localized to the lipid droplet

**Fig. 3:**
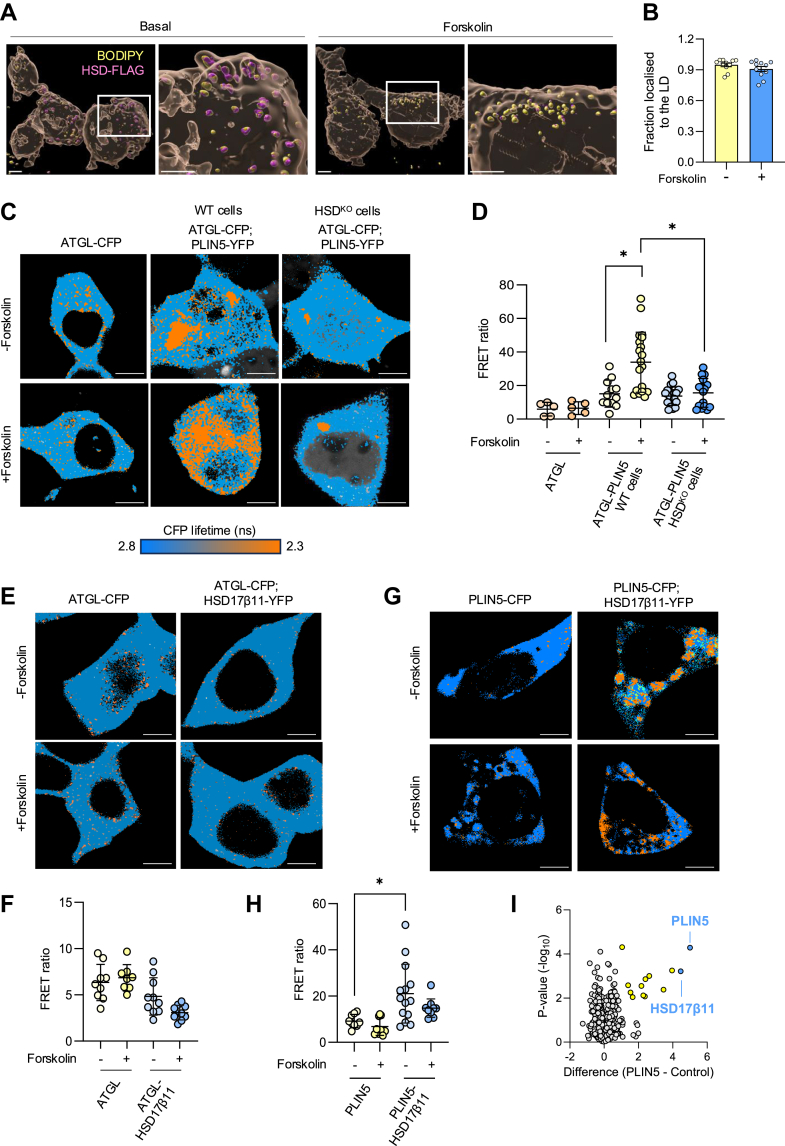
FLIM-FRET analysis reveals HSD17β11 interacts with PLIN5, but not ATGL. A: Representative images of cells showing LDs in yellow (BODIPY 493/503), and Flag-tagged HSD17β11 (⍺Flag) in magenta; channels were merged to show colocalization of LDs and FLAG-tagged protein. All scale bars represent 10 μm. Images were quantified to determine (B) fraction of FLAG localized to the LD (N = 5–6 per group from two independent experiments). C: representative lifetime maps of FLIM acquisition between ATGL-CFP (donor) and PLIN5-YFP (acceptors) pseudo-colored in HSD^KO^ and WT cells in accordance with scale bar (i.e. blue pixels = 0% FRET at 2.8 ns, orange pixels = 19% FRET at 2.3 ns). D: Quantification of the fraction of pixels exhibiting FRET/pixels not exhibiting FRET (i.e. FRET ratio) (N = 5–20 per group from two independent experiments). E: Representative lifetime maps of FLIM acquisition between ATGL-CFP (donor) and HSD17β11-YFP (acceptor). F: Quantification of the fraction of pixels exhibiting FRET/pixels not exhibiting FRET (i.e. FRET ratio). (N = 4–6 per group from two independent experiments). G: Representative lifetime maps of FLIM acquisition between PLIN5-CFP (donor) and HSD17β11-YFP (acceptors) pseudo-colored in. H: Quantification of the fraction of pixels exhibiting FRET/pixels not exhibiting FRET (i.e. FRET ratio). (N = 4–7 per group from two independent experiments). I: Volcano plot of immunoprecipitation analysis of HA-tagged PLIN5. Data are from a label free proteomic analysis of anti-HA immunoprecipitants from cells stabling expressing 3HA-PLIN5 versus 3HA-eGFP. Data is presented as either mean ± SEM (B) or mean ± SD (D, F–H). ∗*P* < 0.05 relative to donor controls as assessed by either unpaired *t* test (B) or one-way ANOVA with Dunnett’s multiple comparisons test (D, F, H). ATGL, adipose triglyceride lipase; FLIM-FRET, fluorescence lifetime imaging-Förster resonance energy transfer; LD, lipid droplet; PLIN5, perilipin 5.

HSD17β11 is a known LD-associated protein (9, 11) and while its ortholog DHS-3 is known to be constitutively bound to LDs (29), whether HSD17β11 itself is constitutive or exchangeable, and whether this function is influenced by β-adrenergic activation (PKA activation) is unknown. To address this question, HSD^KO^ cells were transduced with retrovirus to re-express HSD17β11-FLAG (herein HSD^FLAG^) and were fixed and imaged under basal and forskolin-stimulated conditions. HSD17β11 was constitutively localized to the LD with ∼95% of HSD^FLAG^ localized to the LD under basal and forskolin-stimulated conditions (Fig. 3A–B).

### HSD17β11 interacts with PLIN5 but not ATGL

It is well-recognised that PKA-dependent lipolysis is regulated via the activation of protein-protein complexes (22). Other groups have shown that an HSD17β11 loss-of-function mutation in *C*. *elegans* increased ATGL localization to the surface of the LD (11, 13). This observation raised the possibility that HSD17β11 may regulate lipolysis by coordinating ATGL localization to the LD, perhaps via interactions with PLIN proteins, which are abundantly expressed on LDs and are important regulators of ATGL (22, 30). To test these possibilities, we performed FLIM-FRET analysis in live cells to quantify the interaction between HSD17β11, ATGL, and PLIN5. By using the phasor approach of FLIM-FRET (for detailed methodology, see (23, 31)), we quantified live cell protein interactions between ATGL-CFP (donor) and PLIN5-YFP (acceptor) in HSD^KO^ and WT cells. As we have previously shown (22), ATGL-CFP and PLIN5-YFP have increased FRET efficiency (*i*.*e*. binding affinity) upon PKA-stimulation in WT cells (Fig. 3C, D). ATGL-PLIN5 binding affinity was decreased in HSD^KO^ cells, suggesting that HSD17β11 is required for PKA-mediated co-activation of ATGL by PLIN5.

To determine how HSD17β11 interacts with ATGL and PLIN5, we next quantitated live cell protein interactions between PLIN5-CFP or ATGL-CFP and HSD17β11-YFP at the surface of LDs. Specifically, we examined ATGL-CFP (donor) and HSD17β11-YFP (acceptors). There was no difference in the FRET ratio between ATGL-CFP; HSD17β11-YFP compared to ATGL-CFP only, and this did not change in the presence of forskolin (Fig. 3E, F), confirming there is no direct interaction between HSD17β11 with ATGL.

Analysis of FRET between PLIN5-CFP (donor) and HSD^WT^-YFP (acceptor) revealed a robust interaction between PLIN5 and HSD17β11 (Fig. 3G), as evidenced by a 2.5-fold increase in FRET ratio (Fig. 3H), and this interaction was unaffected with PKA activation. Additional studies using label-free proteomic analysis of anti-HA immunoprecipitants from cells stably expressing 3HA-PLIN5 versus 3HA-eGFP confirmed HSD17β11 interaction with PLIN5 (Fig. 3I). Further anti-FLAG immunoprecipitation from cells stably expressing HSD17β11-FLAG confirmed the PLIN5 interaction via immunoblot (Supplementary Fig. S1). Together, these data demonstrate that PLIN5, but not ATGL, interacts with HSD17β11, and that this interaction occurs independently of PKA stimulation.

Collectively, these data point to a complex role for HSD17β11 in the regulation of lipolysis. HSD17β11 mediates the interaction between ATGL and PLIN5, a known positive regulator of ATGL activity under PKA-stimulated conditions (22). HSD17β11 ablation reduces the interaction between ATGL and PLIN5 under PKA-stimulated conditions, which is associated with reduced lipolysis.

### HSD17β11 regulates lipolysis in human hepatocytes

**Fig. 4:**
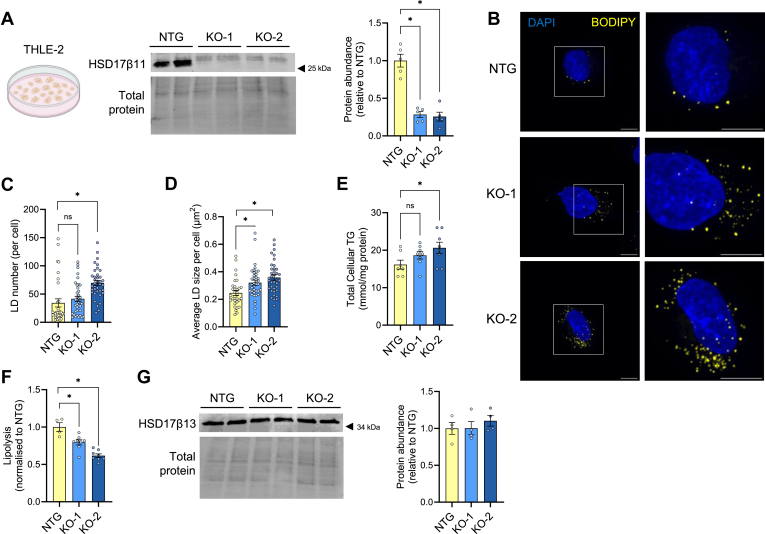
HSD17β11 deletion remodels LD metabolism in THLE-2 cells. A: Representative immunoblot and quantification of non-targeting control (NTG), *HSD17β11* knockout-1 (KO-1) and *HSD17β11* knockout-2 (KO-2) human hepatocyte THLE-2 cells (n = 5 from one experiment) confirming *HSD17β11* knockout. B: Representative confocal images of NTG and KO cells stained for LDs (BODIPY 493/503) and nuclei (DAPI). Images were quantified to determine (C) LD number (count, per cell) and (D) average LD size (μm^2^) (N = 16–19 cells per group from two independent experiments). Scale bars represent 40 μm. E: Total cellular TG. Cells were pulsed with ^14^C-FA for 16h followed by a 6h chase to determine (F) rate of lipolysis (N = 2–4 per group from three independent experiments). (G) Representative immunoblot and quantification of *HSD17β13* abundance in NTG, KO-1 and KO-2 cells (N = 4 from one experiment). Data is presented as mean ± SEM. ∗*P* < 0.05 compared to NTG by one-way ANOVA with Dunnett’s multiple comparisons. DAPI, 4′,6-diamidino-2-phenylindole; KO, knockout; LD, lipid droplet; NTG, non-targeting guide; TG, triglyceride.

Given the association between *HSD17β11* expression and steatosis in human livers, we sought to replicate our key mechanistic findings in cultured human hepatocytes (THLE-2 cells). Two HSD17β11 knockout cell lines (denoted KO-1 and KO-2) were generated using CRISPR-Cas9 with gRNA targeting the first exon of HSD17β11. Immunoblot analysis revealed a 75% and 74% reduction in HSD17β11 protein content in KO-1 and KO-2 respectively, compared to cells transduced with a non-targeting guide (denoted NTG) (Fig. 4A). Ablation of HSD17β11 increased LD number and size and cellular triglycerides compared to NTG (Fig. 4B–E). Additionally, there was a significant decrease in lipolysis (Fig. 4F), phenocopying the changes reported in HEK293T cells (Fig. 2) and confirming that HSD17β11 is required to regulate lipolysis and LD size. Since HSD17β11 and HSD17β13 have a high degree of structural similarity (17), we sought to determine HSD17β13 protein content in HSD17β11 knockout cell lines. Immunoblot analysis revealed no difference in HSD17β13 abundance across all cell lines (Fig. 4G).

### HSD17β11 ablation exerts mild changes in whole-body lipid metabolism in diet-induced obese mice with MASLD

**Fig. 5:**
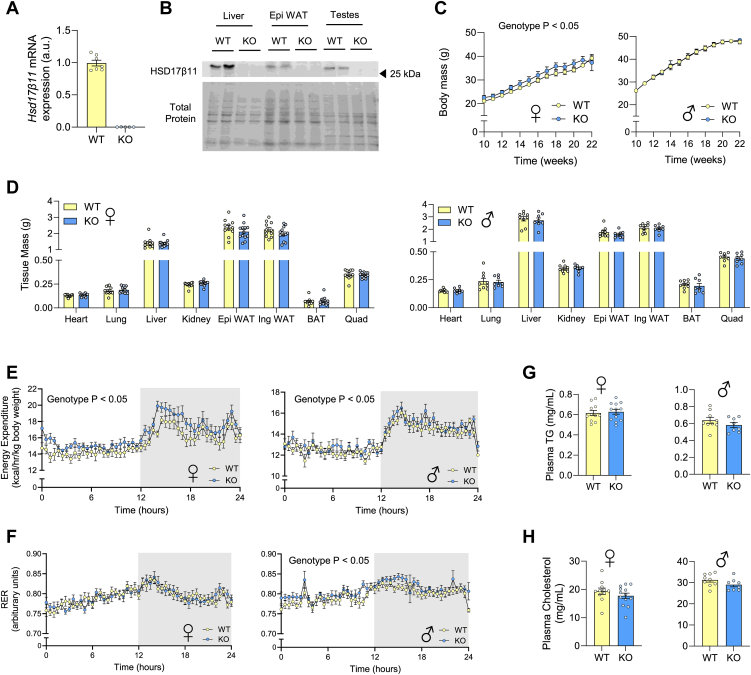
Characterization of Hsd17β11 knockout mice fed an obesogenic diet. A: mRNA expression levels of *Hsd17β11* in wild-type (WT) compared to knockout (KO) mice (N = 5–7 mice). B: Representative immunoblot of HSD17β11 expression in liver, epididymal white adipose tissue (Epi WAT) and testis lysates. C: Weekly body weight of female and male WT and KO mice. At endpoint, key organs were collected and weighed to determine D: tissue mass of lean and adipose tissues. Mice were placed in metabolic cages to determine (E) energy expenditure and (F) respiratory exchange ratio (RER). Blood was collected at endpoint to determine plasma (G) triglyceride (TG) and (H) cholesterol (N = 8–12 per group). Data is presented as mean ± SEM. ∗*P* < 0.05 relative to WT, analysed by unpaired *t* test (A, D, G, H) or two-way ANOVA with Sidak’s multiple comparisons test (C, E, F). BAT, brown adipose tissue; EE, energy expenditure; Epi WAT, epididymal white adipose tissue; Ing WAT, inguinal white adipose tissue; KO, knockout; RER, respiratory exchange ratio; TG, triglyceride; WT, wild-type.

Having shown that HSD17β11 expression is negatively associated with hepatic steatosis in humans with MASLD (Fig. 1) and that HSD17β11 deletion induces lipid accumulation in hepatocytes (Fig. 4), we hypothesized that HSD17β11 is required to prevent hepatic steatosis, especially in diet-induced obesity. To test this hypothesis, we generated HSD17β11 knockout mice using CRISPR-Cas9 gene editing. HSD17β11 deletion was confirmed using qPCR in the liver (Fig. 5A) and immunoblot analysis in several tissues including liver, epididymal white adipose tissue and testis (Fig. 5B).

Since 17β-hydroxysteroid dehydrogenases have a role in sex steroid production and are expressed in both sexes (32), we conducted metabolic phenotyping in both male and female mice. Upon high-fat feeding for 12 weeks, there was a marginal increase in body weight in female HSD17β11^KO^ mice compared with WT (Fig. 5C), whereas no genotype effect was observed in male mice. In line with these mild effects, there was no significant difference in any tissue mass between HSD17β11^KO^ and WT mice for either sex (Fig. 5D). Assessment of whole-body metabolism revealed a minor increase in energy expenditure in HSD17β11^KO^ compared with WT mice for both sexes (Fig. 5E). The respiratory exchange ratio (RER) was not different in female mice and marginally increased in male HSD17β11^KO^ mice, reflecting a subtle increase in whole-body carbohydrate oxidation compared with WT mice (Fig. 5F). Fasting plasma triglyceride and total cholesterol levels were not different between groups (Fig. 5G–H). Taken together, these data demonstrate negligible effects of HSD17β11 deletion on body mass and whole-body substrate oxidation in mice.

### HSD17β11 ablation does not influence lipid metabolism or hepatic steatosis in diet-induced obese mice

**Fig. 6:**
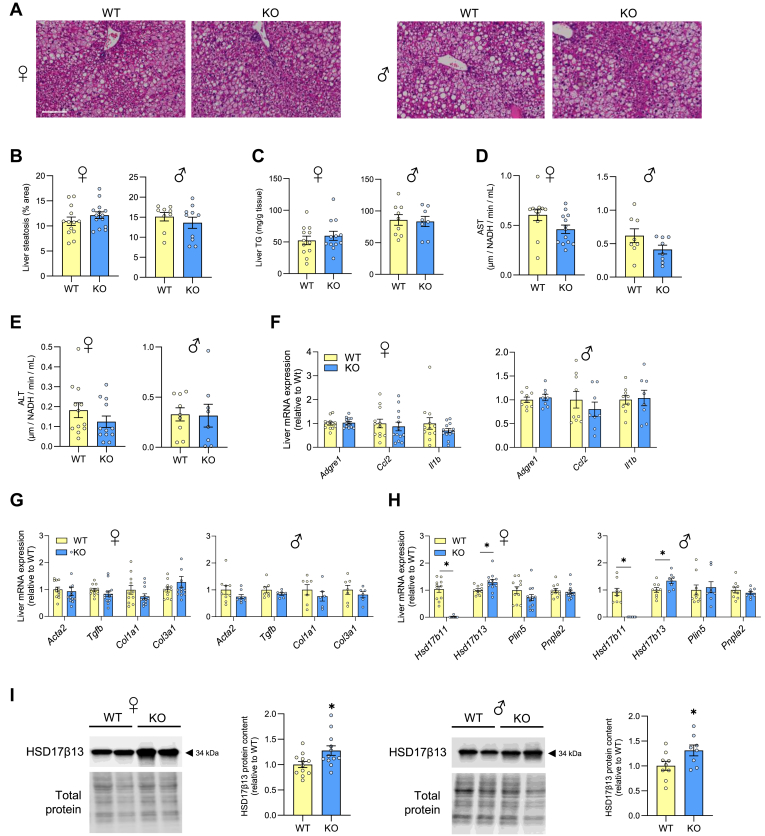
HSD17β11 ablation does not influence liver steatosis or fibrosis in diet-induced obese mice. A: Liver histology of WT and KO mice. Histology sections were quantified as (B) steatosis (as percentage area of section). C: Liver triglyceride (TG). Plasma (D) AST and (E) ALT. Liver mRNA expression of genes that are associated with (F) inflammation, (G) fibrosis and (H) LD proteins. I: Representative immunoblots and quantification of HSD17β13 content in the liver. N = 8–12 per group. Data is presented as mean ± SEM. ∗*P* < 0.05 relative to WT. Data were analysed by unpaired *t* test. Scale bars represent 100 μm. ALT, alanine aminotransferase; AST, aspartate aminotransferase; KO, knockout; TG, triglyceride; WT, wild-type.

Previous studies have shown that HSD17β11 abundance is highly enriched in the liver compared with other tissues (33). This, in conjunction with our characterization of HSD17β11 in regulating lipid metabolism in THLE-2 hepatocytes, led us to consider whether HSD17β11 silencing would affect liver lipid metabolism in mice. Analysis of H&E-stained liver sections (Fig. 6A) revealed no difference in steatosis (Fig. 6B) between livers of HSD17β11^KO^ and WT mice. This was confirmed by biochemical analysis of triglyceride content in livers (Fig. 6C). Consistent with these findings, experiments using radiolabeled fatty acid tracers in precision-cut liver slices revealed no differences between genotypes for liver fatty acid uptake, fatty acid incorporation into various lipid species, or fatty acid oxidation (Supplementary Fig. S2).

There were no differences in the liver damage markers AST and ALT (Fig. 6D–E) or genes associated with liver inflammation (*Adgre1*, *Ccl2*, *Il1b*) and fibrosis (*Acta2*, *Tgfb*, *Col1a1*, *Col3a1*) (Fig. 6F–G). While there were no changes in the mRNA expression of key regulatory enzymes of lipolysis, including *Plin5* and *Pnpla2* (gene encoding ATGL), there was an increase in *Hsd17β13* mRNA expression in both female and male HSD17β11^KO^ mice compared with WT (Fig. 6H). Aligning with the mRNA levels, HSD17β13 protein content was increased by ∼30% in female and male HSD17β11^KO^ mice (Fig. 6I). Collectively, these data demonstrate that HSD17β11 deletion does not accelerate high-fat diet-induced MASLD in mice. The findings from these experiments were recapitulated in mice fed a low-fat chow diet (Supplementary Fig. S3). Taken together, these data suggest that HSD17β11 is redundant for hepatic lipid metabolism in mice, perhaps due to a compensatory increase in HSD17β13.

## Discussion

LDs maintain cellular lipid homeostasis via the release and storage of fatty acids. These processes are orchestrated by PKA-mediated phosphorylation to induce translocation and docking of proteins to facilitate protein–protein interactions and lipolysis. In this study, we identified HSD17β11 as a regulator of LD metabolism that facilitates lipolysis by modulating interactions between ATGL and PLIN5 in human liver cells.

Although several studies have explored the cellular and molecular roles of 17β-hydroxysteroid dehydrogenases, the specific mechanism by which HSD17β11 regulates LD metabolism remains largely uncharacterized. A previous study in *C*. *elegans* reported that loss-of-function mutations in an ortholog of HSD17β11 (DHS-3) resulted in reduced triglyceride content and smaller LDs (11). In contrast, our findings demonstrate that deletion of HSD17β11 in human cells (HEK293 T and THLE-2) leads to a marked increase in triglyceride level, as well as an increase in LD size and number. These contrasting observations highlight a possible functional divergence of 17β-HSD family members across species and between paralogs, despite conserved structural features and catalytic motifs (10). Using metabolic tracer approaches, we show that HSD17β11 deficiency enhances fatty acid uptake and esterification, promoting the accumulation of neutral lipid species, including cholesterol esters and phospholipids, all of which are key components of LDs. The mechanism underpinning this effect on fatty acid uptake is elusive, as the majority of HSD17β11 is localized to intracellular LDs. Importantly, lipolysis was significantly reduced in HSD^KO^ cells, demonstrating that HSD17β11 is required for efficient lipolysis in human cells.

HSD17β11 contains a PAT-like motif in its N-terminal domain that is present in PLIN proteins and is used to localize the protein to the LD surface (12). In line with previous studies, we show that ∼95% of HSD17β11 is localized to the LD in both basal and PKA-stimulated states, supporting the notion that HSD17β11 actions occur principally at the LD (4, 34). Interestingly, unlike ATGL and other lipolytic proteins, forskolin stimulation of PKA does not affect HSD17β11 localization to the LD.

PKA phosphorylation of lipolytic proteins regulates various functions, including catalytic activity (*e*.*g*., HSL, ATGL), translocation to and from the LD (*e*.*g*., HSL, PLIN5), and altered binding affinity of proteins to promote lipolysis (*e*.*g*., ABHD5 with ATGL) (22, 35, 36, 37, 38, 39). Using FLIM-FRET and immunoprecipitation-MS/MS approaches in cells, we extend on this knowledge by showing that HSD17β11 interacts with PLIN5, but that this interaction is not enhanced with PKA-simulation. Rather, HSD17β11 is required to facilitate the interaction of PLIN5 and ATGL to permit efficient PKA-stimulated lipolysis. Notably, HSD17β11 regulation of lipolysis differs from HSD17β13, which facilities lipolysis when phosphorylated by PKA on serine 33 which promotes its interaction with ATGL (28). These new observations align with studies ascribing novels’ roles of other LD-associated HSD family members. For example, pharmacological inhibition of HSD17β1 improves body weight, HbA1c levels and insulin sensitivity in patients with Type 2 diabetes (40, 41), HSD17β2 depletion in enterocytes increases triglyceride secretion (42), while liver-specific knockout of HSD17β12 leads to weight loss, reduced adipose LD size and increased liver triglyceride in mice (43). This suggests that beyond their classical enzymatic roles in steroid metabolism, 17β-HSDs have important and varied roles in maintaining other aspects of lipid metabolism (10).

Whole-body HSD17β11 deletion in mice did not impact fatty acid metabolism in the liver or hepatic steatosis, and exerted few phenotypic changes, which included a very modest increase in body mass and energy expenditure in female mice and a minor increase in carbohydrate oxidation in male mice. This was unexpected since HSD17β11 is downregulated in humans with MASLD and HSD17β11 deletion reduced lipolysis and increased LD accumulation and triglyceride storage in human cells. There are several possibilities to explain this apparent discrepancy. Others have shown that inactivating mutations in HSD17β13 protect humans from MASLD (14, 44), yet *Hsd17β13* deficiency in mice did not reproduce the protective role of HSD17β13 loss-of-function mutants (27). Analogous to this scenario for HSD17β13, it is possible that HSD17β11 functions in humans are not faithfully recapitulated in mice. Second, it is possible that the loss of HSD17β11 is compensated for by an increase in HSD17β13 (30% in both sexes). Notably, when HSD17β13 was not increased with HSD17β11 deletion in THLE-2 cells, lipolysis was reduced, which supports the possibility of a compensatory effect HSD17β13 in regulating lipolysis in vivo. In this regard, HSD17β13 and HSD17β11 share conserved features including homodimer interaction, cofactor binding, substrate binding and catalytic sites (44). However, there are notable differences, for example, HSD17β11 binds to PLIN5 but not ATGL, whereas HSD17β13 does not bind to PLIN5 but binds to ATGL (28). Additionally, HSD17β11 and HSD17β13 expression within liver cells vary (Supplementary Fig. S4): HSD17β11 is expressed in hepatocytes, endothelial cells, stromal cells, and many immune cells including neutrophils, basophils, resident NK cells, T cells, macrophages, monocytes, and cholangiocytes, whilst HSD17β13 is almost exclusively expressed in hepatocytes. Further, HSD17β11 expression does not change in obesity, whereas HSD17β13 expression is decreased in obese individuals (45). While this indicates the possibility of similar substrate preferences and functional roles, the mechanisms by which these 17β-HSD family members regulate lipolysis are likely to be divergent and this requires further examination.

In summary, our study demonstrates a role for HSD17β11 in regulating lipid metabolism in cells. We show that HSD17β11 is almost exclusively localized to the LD and interacts with PLIN5 to facilitate PLIN5-ATGL interactions to promote efficient lipolysis. While this process occurs in human cells, HSD17β11 is not essential for the control of lipid metabolism in mice. This may reflect a species difference between the biological functions of HSD17β11 in humans and mice, as is the case between human cells and *C*. *elegans*, or possible compensation by HSD17β13 in regulating LD metabolism in the absence of HSD17β11.

## Data availability

The authors declare that all data is contained in the manuscript.

### Supplemental data

This article contains supplemental data.

### Declaration of generative AI and AI-assisted technologies

The authors declare no use of generative AI or AI-assisted technologies.

## Conflict of interest

The authors declare that they do not have any conflicts of interest with the content of this article.

## References

[1] Olzmann J.A., Carvalho P. (2019). Dynamics and functions of lipid droplets. Nat. Rev. Mol. Cell Biol.

[2] Kory N., Farese R.V., Walther T.C. (2016). Targeting fat: mechanisms of protein localization to lipid droplets. Trends Cell Biol..

[3] Bersuker K., Peterson C.W.H., To M., Sahl S.J., Savikhin V., Grossman E.A. (2018). A proximity labeling strategy provides insights into the composition and dynamics of lipid droplet proteomes. Dev. Cell.

[4] Zhang C., Liu P. (2019). The new face of the lipid droplet: lipid droplet proteins. Proteomics.

[5] Bezawork-Geleta A., Devereux C.J., Keenan S.N., Lou J., Cho E., Nie S. (2025). Proximity proteomics reveals a mechanism of fatty acid transfer at lipid droplet-mitochondria- endoplasmic reticulum contact sites. Nat. Commun..

[6] Mejhert N., Gabriel K.R., Frendo-Cumbo S., Krahmer N., Song J., Kuruvilla L. (2022). The lipid droplet knowledge portal: a resource for systematic analyses of lipid droplet biology. Dev. Cell.

[7] Fujimoto Y., Itabe H., Sakai J., Makita M., Noda J., Mori M. (2004). Identification of major proteins in the lipid droplet-enriched fraction isolated from the human hepatocyte cell line HuH7. Biochim. Biophys. Acta.

[8] Liu P., Ying Y., Zhao Y., Mundy D.I., Zhu M., Anderson R.G.W. (2004). Chinese hamster ovary K2 cell lipid droplets appear to be metabolic organelles involved in membrane traffic. J. Biol. Chem..

[9] Yu J., Zhang L., Li Y., Zhu X., Xu S., Zhou X.M. (2018). The adrenal lipid droplet is a new site for steroid hormone metabolism. Proteomics.

[10] Liang B., Fu L., Liu P. (2024). Regulation of lipid droplet dynamics and lipid homeostasis by hydroxysteroid dehydrogenase proteins. Trends Cell Biol..

[11] Liu Y., Xu S., Zhang C., Zhu X., Hammad M.A., Zhang X. (2018). Hydroxysteroid dehydrogenase family proteins on lipid droplets through bacteria, C elegans, and mammals. Biochim. Biophys. Acta Mol. Cell Biol Lipids.

[12] Horiguchi Y., Araki M., Motojima K. (2008). Identification and characterization of the ER/lipid droplet-targeting sequence in 17beta-hydroxysteroid dehydrogenase type 11. Arch. Biochem. Biophys..

[13] Thomes P.G., Strupp M.S., Donohue T.M., Kubik J.L., Sweeney S., Mahmud R. (2023). Hydroxysteroid 17β-dehydrogenase 11 accumulation on lipid droplets promotes ethanol-induced cellular steatosis. J. Biol. Chem..

[14] Abul-Husn N.S., Cheng X., Li A.H., Xin Y., Schurmann C., Stevis P. (2018). A protein-truncating HSD17B13 variant and protection from chronic liver disease. N. Engl. J. Med..

[15] Su W., Wang Y., Jia X., Wu W., Li L., Tian X. (2014). Comparative proteomic study reveals 17beta-HSD13 as a pathogenic protein in nonalcoholic fatty liver disease. Proc. Natl. Acad. Sci. U. S. A..

[16] Liu S., Huang C., Li D., Ren W., Zhang H., Qi M. (2007). Molecular cloning and expression analysis of a new gene for short-chain dehydrogenase/reductase 9. Acta Biochim. Pol..

[17] Ma Y., Karki S., Brown P.M., Lin D.D., Podszun M.C., Zhou W. (2020). Characterization of essential domains in HSD17B13 for cellular localization and enzymatic activity. J. Lipid Res..

[18] Brunt E.M., Kleiner D.E., Wilson L.A. (2011). Nonalcoholic fatty liver disease activity score and the histopathologic diagnosis in NAFLD: distinct clinicopathologic meanings. Hepatology.

[19] European Association for the Study of the Liver (EASL), European Association for the Study of Diabetes (EASD), European Association for the Study of Obesity (EASO) (2016). EASL-EASD-EASO Clinical Practice Guidelines for the management of non-alcoholic fatty liver disease. J Hepatol.

[20] Fidelito G., Todorovski I., Cluse L., Vervoort S.J., Taylor R.A., Watt M.J. (2025). Lipid-metabolism-focused CRISPR screens identify enzymes of the mevalonate pathway as essential for prostate cancer growth. Cell Rep.

[21] De Nardo W., Miotto P.M., Bayliss J., Nie S., Keenan S.N., Montgomery M.K., Watt M.J. (2022). Proteomic analysis reveals exercise training induced remodelling of hepatokine secretion and uncovers syndecan-4 as a regulator of hepatic lipid metabolism. Mol. Metab..

[22] Keenan S.N., De Nardo W., Lou J., Schittenhelm R.B., Montgomery M.K., Granneman J.G. (2021). Perilipin 5 S155 phosphorylation by PKA is required for the control of hepatic lipid metabolism and glycemic control. J. Lipid Res..

[23] Lou J., Scipioni L., Wright B.K., Bartolec T.K., Zhang J., Masamsetti V.P. (2019). Phasor histone FLIM-FRET microscopy quantifies spatiotemporal rearrangement of chromatin architecture during the DNA damage response. Proc. Natl. Acad. Sci. U. S. A..

[24] Liang Z., Lou J., Scipioni L., Gratton E., Hinde E. (2020). Quantifying nuclear wide chromatin compaction by phasor analysis of histone forster resonance energy transfer (FRET) in frequency domain fluorescence lifetime imaging microscopy (FLIM) data. Data Brief.

[25] Keenan S.N., Meex R.C., Lo J.C.Y., Ryan A., Nie S., Montgomery M.K. (2019). Perilipin 5 Deletion in Hepatocytes Remodels Lipid Metabolism and Causes Hepatic Insulin Resistance in Mice. Diabetes.

[26] Berg S., Kutra D., Kroeger T., Straehle C.N., Kausler B.X., Haubold C. (2019). ilastik: interactive machine learning for (bio)image analysis. Nat Methods.

[27] Ma Y., Brown P.M., Lin D.D., Ma J., Feng D., Belyaeva O.V. (2021). 17-Beta hydroxysteroid dehydrogenase 13 deficiency does not protect mice from obesogenic diet injury. Hepatology.

[28] Su W., Wu S., Yang Y., Guo Y., Zhang H., Su J. (2022). Phosphorylation of 17beta-hydroxysteroid dehydrogenase 13 at serine 33 attenuates nonalcoholic fatty liver disease in mice. Nat. Commun..

[29] Zhang P., Na H., Liu Z., Zhang S., Xue P., Chen Y. (2012). Proteomic study and marker protein identification of Caenorhabditis elegans lipid droplets. Mol. Cell Proteomics.

[30] Granneman J.G., Moore H.P.H., Mottillo E.P., Zhu Z., Zhou L. (2011). Interactions of perilipin-5 (Plin5) with adipose triglyceride lipase. J. Biol. Chem..

[31] Hinde E., Digman M.A., Welch C., Hahn K.M., Gratton E. (2012). Biosensor forster resonance energy transfer detection by the phasor approach to fluorescence lifetime imaging microscopy. Microsc. Res. Tech..

[32] Saloniemi T., Jokela H., Strauss L., Pakarinen P., Poutanen M. (2012). The diversity of sex steroid action: novel functions of hydroxysteroid (17beta) dehydrogenases as revealed by genetically modified mouse models. J. Endocrinol..

[33] Yokoi Y., Horiguchi Y., Araki M., Motojima K. (2007). Regulated expression by PPARalpha and unique localization of 17beta-hydroxysteroid dehydrogenase type 11 protein in mouse intestine and liver. FEBS J..

[34] Brasaemle D.L., Dolios G., Shapiro L., Wang R. (2004). Proteomic analysis of proteins associated with lipid droplets of basal and lipolytically stimulated 3T3-L1 adipocytes. J. Biol. Chem..

[35] Marcinkiewicz A., Gauthier D., Garcia A., Brasaemle D.L. (2006). The phosphorylation of serine 492 of perilipin a directs lipid droplet fragmentation and dispersion. J. Biol. Chem..

[36] Mason R.R., Meex R.C.R., Lee-Young R., Canny B.J., Watt M.J. (2012). Phosphorylation of adipose triglyceride lipase Ser(404) is not related to 5'-AMPK activation during moderate-intensity exercise in humans. Am. J. Physiol. Endocrinol. Metab..

[37] Pagnon J., Matzaris M., Stark R., Meex R.C.R., Macaulay S.L., Brown W. (2012). Identification and functional characterization of protein kinase A phosphorylation sites in the major lipolytic protein, adipose triglyceride lipase. Endocrinology.

[38] Sahu-Osen A., Montero-Moran G., Schittmayer M., Fritz K., Dinh A., Chang Y.F. (2015). CGI-58/ABHD5 is phosphorylated on Ser239 by protein kinase A: control of subcellular localization. J. Lipid Res..

[39] Watt M.J., Holmes A.G., Pinnamaneni S.K., Garnham A.P., Steinberg G.R., Kemp B.E., Febbraio M.A. (2006). Regulation of HSL serine phosphorylation in skeletal muscle and adipose tissue. Am. J. Physiol. Endocrinol. Metab..

[40] Feig P.U., Shah S., Hermanowski-Vosatka A., Plotkin D., Springer M.S., Donahue S. (2011). Effects of an 11beta-hydroxysteroid dehydrogenase type 1 inhibitor, MK-0916, in patients with type 2 diabetes mellitus and metabolic syndrome. Diabetes Obes. Metab..

[41] Rosenstock J., Banarer S., Fonseca V.A., Inzucchi S.E., Sun W., Yao W. (2010). The 11-beta-hydroxysteroid dehydrogenase type 1 inhibitor INCB13739 improves hyperglycemia in patients with type 2 diabetes inadequately controlled by metformin monotherapy. Diabetes Care.

[42] Beilstein F., Bouchoux J., Rousset M., Demignot S. (2013). Proteomic analysis of lipid droplets from Caco-2/TC7 enterocytes identifies novel modulators of lipid secretion. PLoS One.

[43] Heikela H., Ruohonen S.T., Adam M., Viitanen R., Liljenbäck H., Eskola O. (2020). Hydroxysteroid (17beta) dehydrogenase 12 is essential for metabolic homeostasis in adult mice. Am. J. Physiol. Endocrinol. Metab..

[44] Ma Y., Belyaeva O.V., Brown P.M., Fujita K., Valles K., Karki S. (2019). 17-Beta hydroxysteroid dehydrogenase 13 is a hepatic retinol dehydrogenase associated with histological features of nonalcoholic fatty liver disease. Hepatology.

[45] Guilliams M., Bonnardel J., Haest B., Vanderborght B., Wagner C., Remmerie A. (2022). Spatial proteogenomics reveals distinct and evolutionarily conserved hepatic macrophage niches. Cell.

