# Supplementary figures and images for: HSD17β11 regulates PLIN5-ATGL mediated lipolysis, but not hepatic lipid metabolism in mice
Source: J Lipid Res. 2025 Nov 12;66(12):100943. doi: 10.1016/j.jlr.2025.100943 (PMC12743511; doi:10.1016/j.jlr.2025.100943)

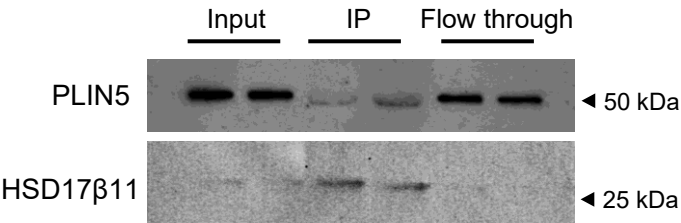

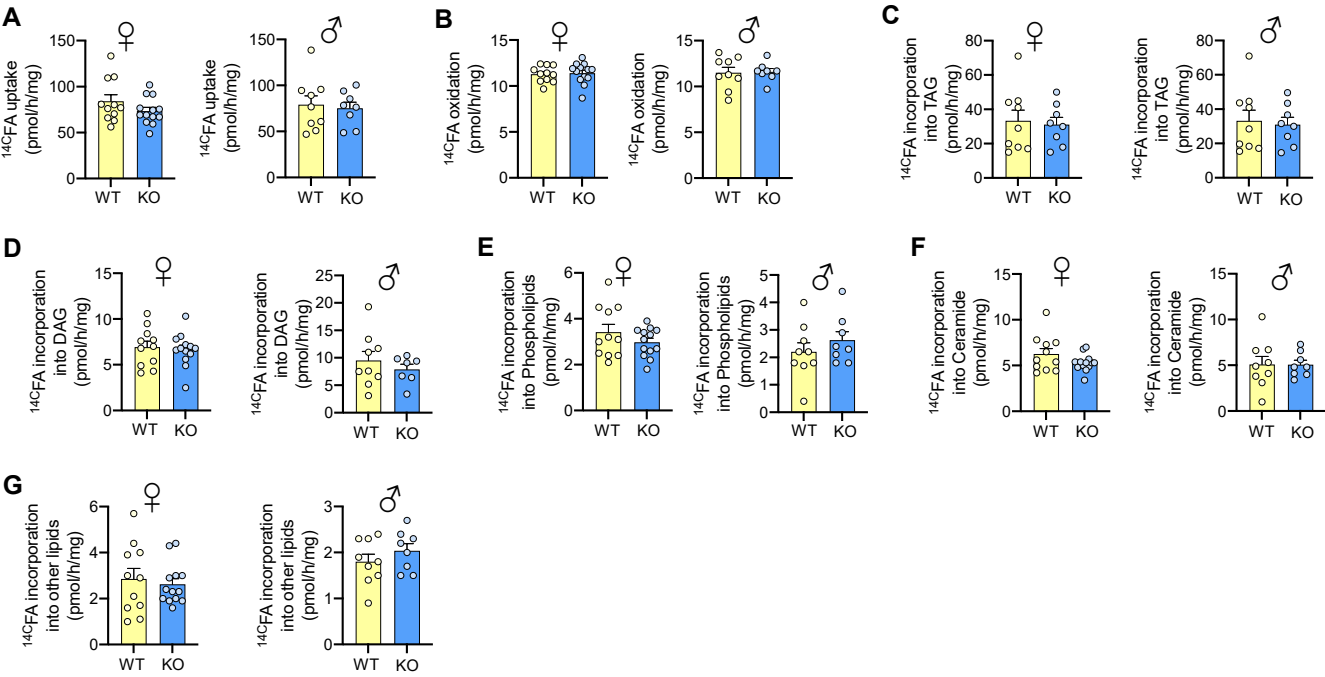

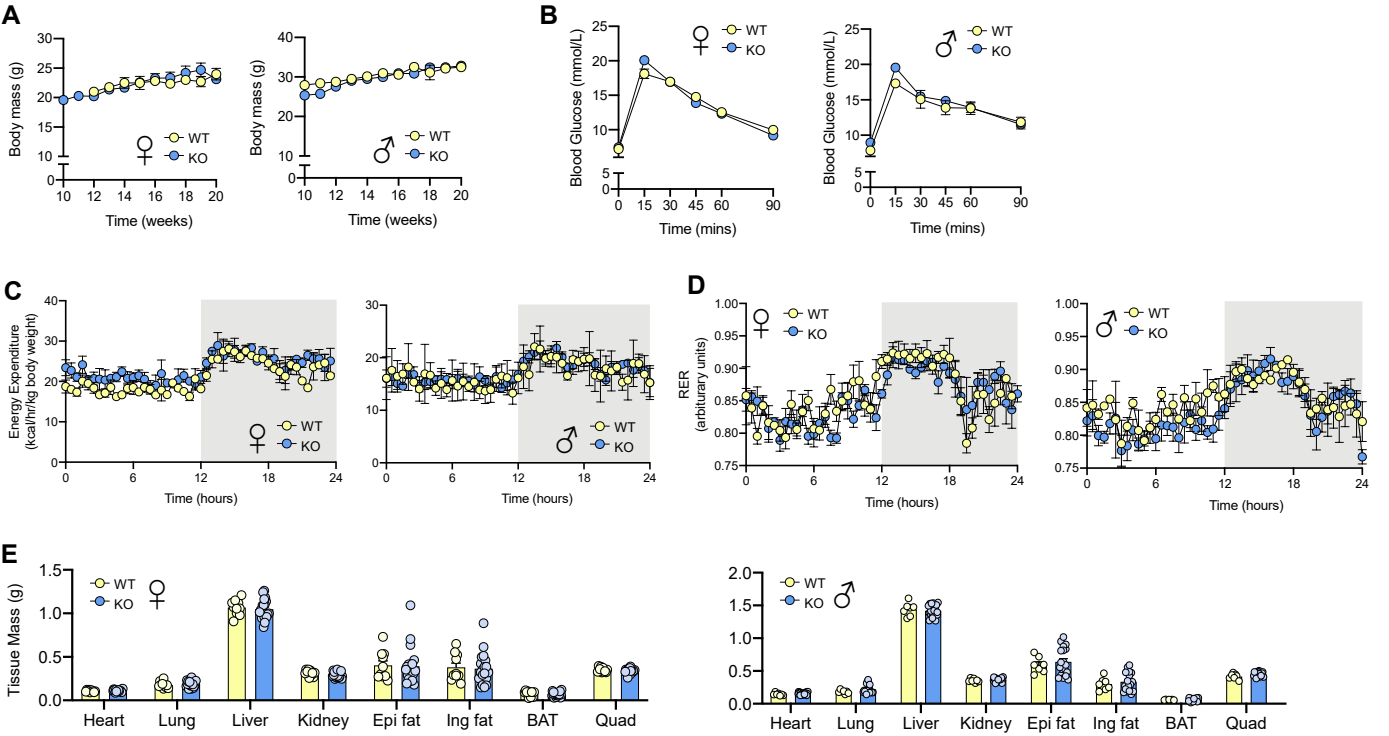

A

HSD17β11

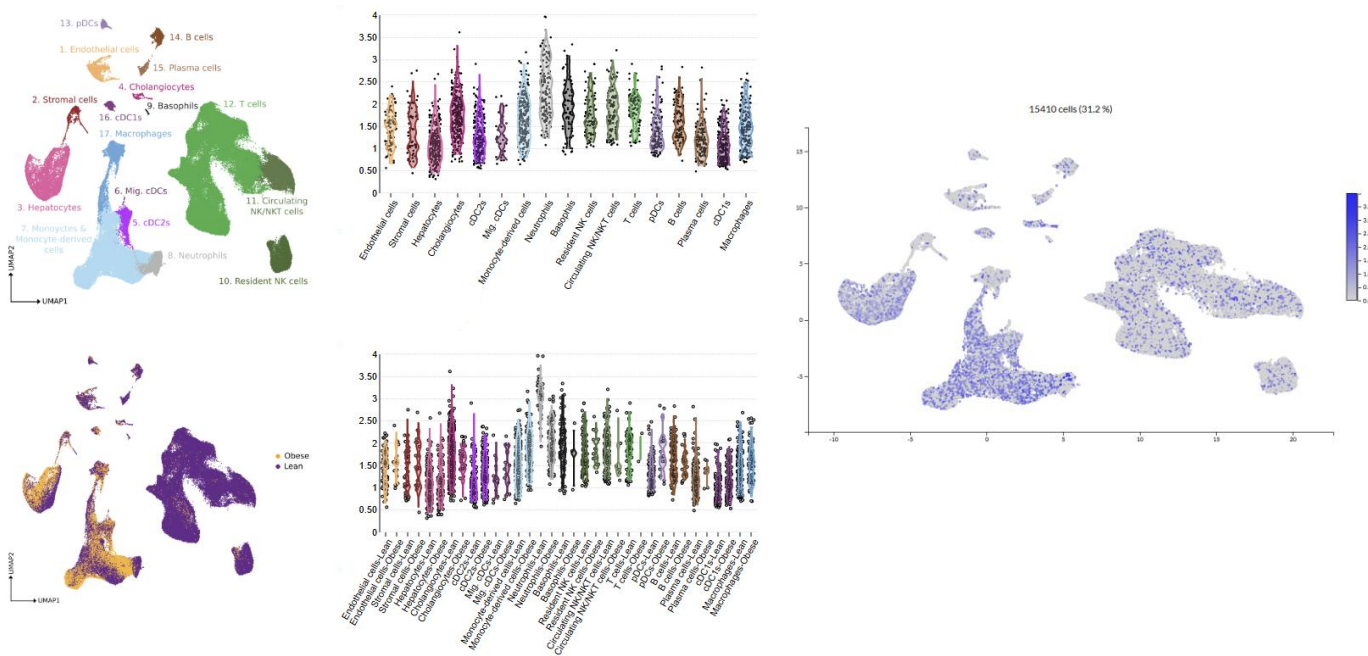

B

HSD17β13

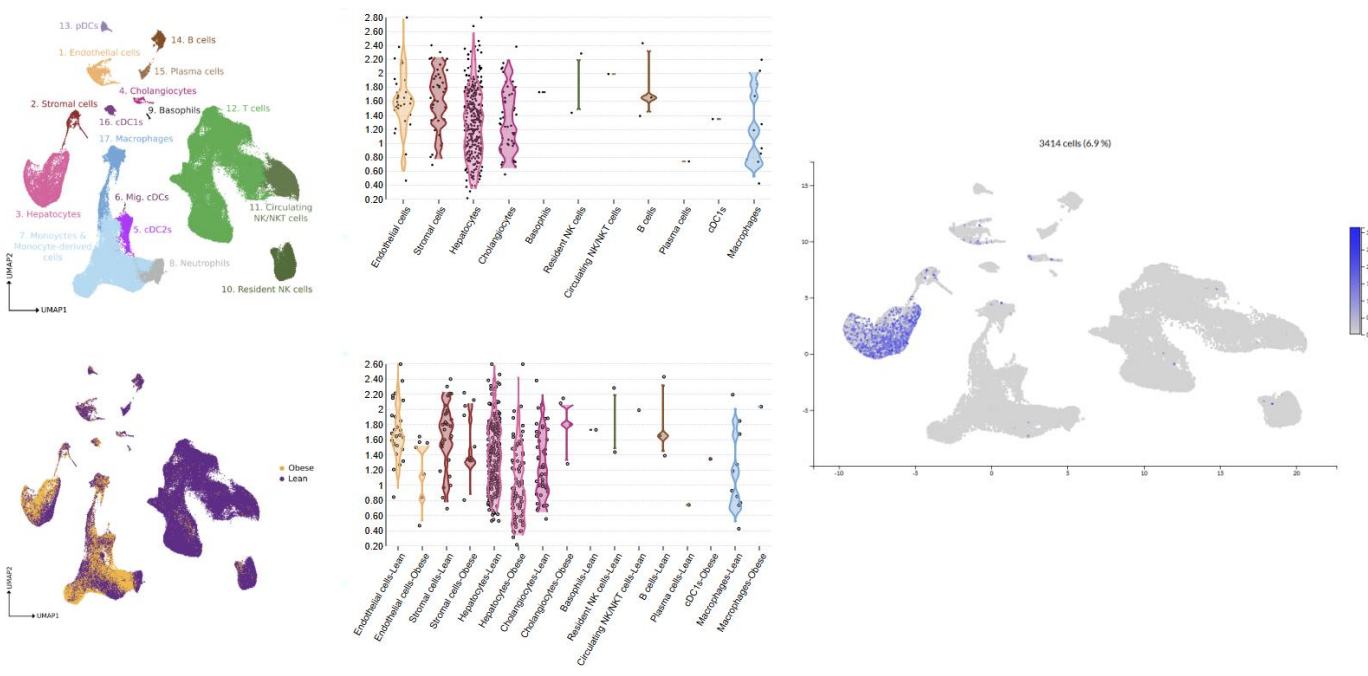

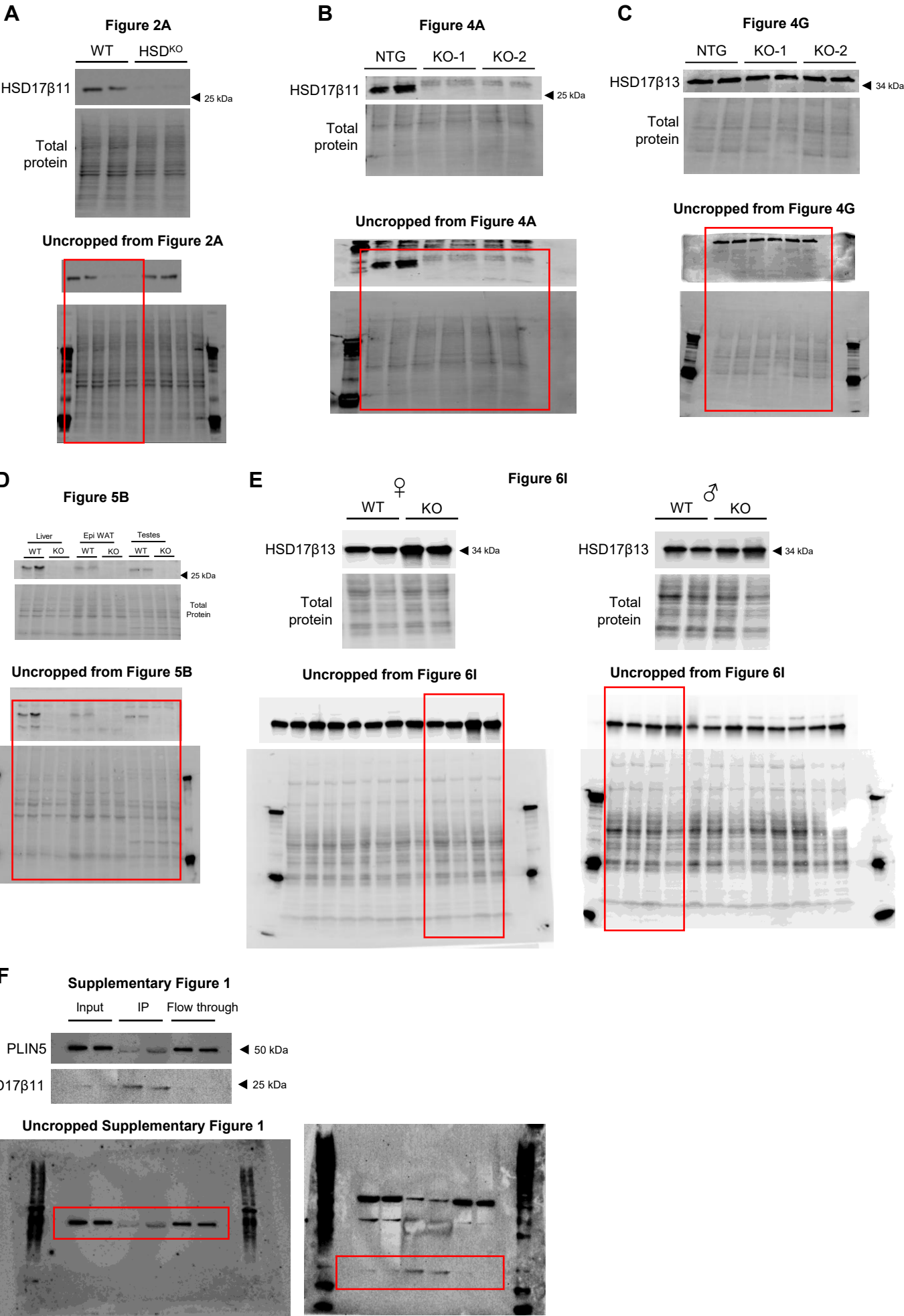

Supplement: Supplemental Figures [file mmc1.pdf]
